# Bioluminescent Whole-Cell Bioreporter Bacterial Panel for Sustainable Screening and Discovery of Bioactive Compounds Derived from Mushrooms

**DOI:** 10.3390/bios14110558

**Published:** 2024-11-17

**Authors:** Calin Trif, Jovana Vunduk, Yardnapar Parcharoen, Aporn Bualuang, Robert S. Marks

**Affiliations:** 1 Avram and Stella Goldstein-Goren Department of Biotechnology Engineering, Faculty of Engineering Sciences, Ben-Gurion University of Negev, Beer Sheva 84105, Israel; trif@post.bgu.ac.il; 2 Institute of General and Physical Chemistry, Studentski trg 12/V, 11158 Belgrade, Serbia; 3 Chulabhorn International College of Medicine, Thammasat University, Pathum Thani 12120, Thailand; yardnapa@tu.ac.th (Y.P.);

**Keywords:** antimicrobial activity, bioluminescence, medicinal mushrooms, toxicity, whole cell bioreporter bacteria, quorum sensing

## Abstract

This study presents a rapid and comprehensive method for screening mushroom extracts for the putative discovery of bioactive molecules, including those exhibiting antimicrobial activity. This approach utilizes a panel of bioluminescent bacteria, whose light production is a sensitive indicator of various cellular effects triggered by the extracts, including disruption of bacterial communication (quorum sensing), protein and DNA damage, fatty acid metabolism alterations, and oxidative stress induction. The bioassay’s strength is its ability to efficiently analyze a large number of extracts simultaneously while also assessing several different mechanisms of toxicity, significantly reducing screening time. All samples analyzed exhibited more than one cellular effect, as indicated by the reporter bacteria. Four samples (*C. cornucopioides*, *F. fomentarius*, *I. obliquus*, and *M. giganteus*) displayed the highest number (six) of possible mechanisms of antibacterial activity. Additionally, combining extraction and purification protocols with a bioluminescent bacterial panel enables simultaneous improvement of the desired antimicrobial properties of the extracts. The presented approach offers a valuable tool for uncovering the diverse antimicrobial mechanisms of mushroom extracts.

## 1. Introduction

Some 1513 bacterial species are known as human pathogens, whose fast mutations enable them to develop antibiotic resistance, thus representing a constant and perpetual threat [1]. This is compounded by the accelerating use of antibiotics in China, India, Russia, South Africa, and Brazil, whose populations are increasing [2,3]. We live in an era of the antibiotic resistance crisis, whose predicament may be compounded by the increasingly problematic “climate change” [3,4]. 

On one hand, the World Health Organization developed an antibiotic resistance action plan that includes an increased supply of novel antibiotics [5]. At the same time, pharmaceutical companies are not investing in the search for early candidates (so-called “hit substances”) with bacteriostatic activity due to the investment involved. Moreover, as Miethke et al. [5] stated, the cost of new scaffold discovery is much higher than derivatizing existing ones. This, together with antibiotic overuse and self-medication, created a continued need to search for novel antimicrobial compounds. Whoever embarks on the lengthy and costly discovery of hit bioactive molecules, including antimicrobials [6], requires novel, versatile, high-throughput methods, providing speed, reliability, and reproducibility at affordable costs.

The bottleneck in a discovery pipeline is the initial screening since it requires a lot of time and resources, especially if traditional cultivation techniques are involved. Moreover, non-secreted natural compounds are always present in a complex matrix, which makes their identification and mechanism of action a tedious task. This is also true when natural compounds exhibit a combinatory effect [7]. Traditional methods for assessing antimicrobial activity are the dominant ones, with such disadvantages as low reproducibility and lengthy and sometimes tedious protocols, while providing no insight into the mechanism of antibacterial action. Currently used techniques include the disc-diffusion method, dilution (both macro and micro), Etest, MALDI-TOF MS, and PCR. All cited methods have distinct disadvantages, like poor performance when analyzing slow-growing bacteria, the influence of physiochemical factors, low reproducibility and sensitivity, need for semi-automation, high variability of the results, false positive results, long incubation time, tedious protocol steps, high chance of cross-contamination, the inability to differentiate between viable and nonviable bacterial cells, maintenance of the optimal physiochemical conditions, expensive batch performance, the inaccurate and inconsistent behavior of some antibacterial agents, a complicated and expensive laboratory set-up, instrument maintenance, and high cost of reagents and instruments [8]. Emerging techniques like microfluidics-based diagnostics, electrochemical devices, different optical sensors, or ATP bioluminescence assays are characterized by small sample volume, reproducibility, high level of work environment control, portability, speed, and cost-effectiveness [9]. However, these have limitations in their applicability of natural extracts.

Live whole-cell bioreporters have the ability to screen their environment for harmful compounds and have the additional capability to provide both a visible and quantifiable reaction. We have demonstrated that these systems could be explored and tailored to provide fast and reliable measurements of water toxicity [10] (such as heavy metals [11], pesticides [12], sediments [13], pathogens [14], and carcinogens [15]) and other toxicity-inducing compounds (degradation of remediated toxicants [16], cigarette toxicants [17], and estrogens [18]). Furthermore, these bioreporters were also used for the detection of antibiotics [19], undiscovered antibiotics [20], artificial sweeteners [21], nanomaterials [22] (including carbon nanotubes [23]), cannabis compounds [24], and air pollution [25]. They are used in a variety of formats, including fiber-optic biosensors [26], liquid-light guide biosensors [27], flow-through systems [28], river on-line monitoring [29], whose sensitivity may be modulated with metal-enhanced bioluminescence [30] and other protocols [31]. The use of such systems is not limited to the laboratory only and can be used in the field as well [32]. Other studies have been conducted in medical fields [33] and on the improvement of such biological systems [34]. Therefore, these bioreporters are an ideal platform for screening toxicants, including antimicrobial compounds [35]. Specific promoter–reporter gene constructs, such as *luxCDABE* operons, that respond to target analytes or environmental conditions are incorporated in these engineered bacterial cells and react to the presence of specific toxic compounds in their vicinity. Moreover, setting up a panel of bioreporter bacteria enables a real-time, fast, and quantifiable response to an unknown ‘target’ compound, including its putative mechanism of antimicrobial action. By transferring this system to a microtiter plate in an in vitro setting, using the same logic as the common antimicrobial activity tests, some hurdles of traditional methods can be overcome. Moreover, as mentioned before, the results of this bioassay can provide invaluable early insight into the possible mechanism of the active compounds’ action. The induction or suppression of bioluminescence in those bioreporters when exposed to toxicants typically occurs through direct interaction with regulatory proteins, activation of stress–response pathways, or interference with cellular metabolic processes, leading to changes in gene expression that ultimately affect light production. This prospect is of great value for testing complex sources of antimicrobials like naturally derived extracts.

Currently, two-thirds of all antimicrobials in use (for healthcare, agriculture, veterinary, and food purposes) are derived from natural resources. It is interesting to point out that current research is returning to prioritize the discovery of new antibiotics (and other bioactive compounds) through a quest for natural sources, particularly in the world of macrofungi, reflecting the past (1940s–60s) microfungal antibiotic sources [36]. The recent search for “fungiceuticals” produced a number of promising candidates, including calvatic acid, β-metoxyiacrilic acid derivatives, strobilurins, oudemansins, scorodonin, pterulones, pterulinic acid, and merulidial, while so far, only pleuromutilin was approved for human use [36]. The same authors provided a review of mushroom-derived compounds effective against superbugs, bacteria that are highly resistant to existing antibiotics, like methicillin-resistant *Staphylococcus aureus* (MRSA). They point to a significant gap in species examined for antimicrobial activity and our current understanding of their active compounds’ mechanism(s) of action [37].

**Figure 1:**
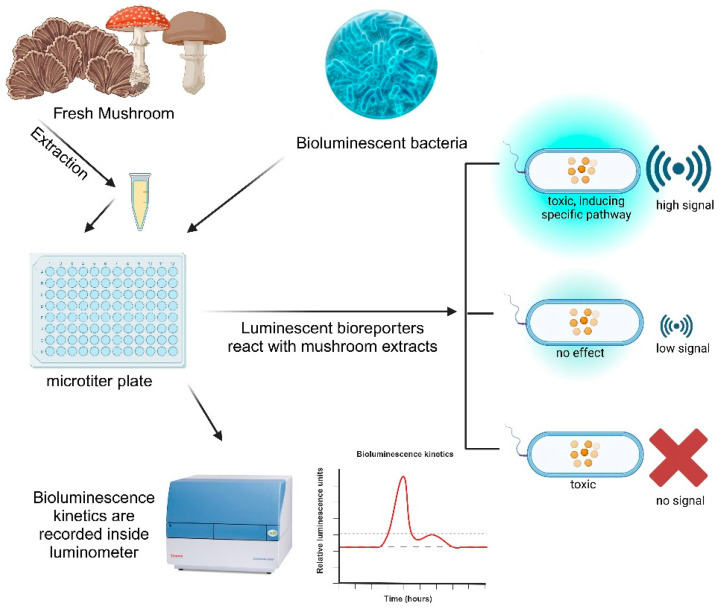
Workflow of the protocol and interpretation of results. Mushroom extracts react with bioluminescent bacteria inside microtiter plates and the reaction is recorded inside a luminometer. These mutant strains produce a low signal in a stress-free environment, and their increase or decrease in bioluminescence is an indicator of toxicity. (Figure created in BioRender.com) [38].

The present study aims to test the prospects of using the aforementioned bioreporter whole-cell bacterial panel for the fast and reproducible identification of antimicrobial compounds (Figure 1). Taking into consideration the actuality of mushrooms as a potential source of novel drug candidates, 39 mushroom-derived extracts were tested using the proposed system. In addition, the goal was to demonstrate that the bioreporter whole-cell bacterial panel can not only identify which extract is active and at which concentration but also, at the same time, point to a possible mechanism of toxicity. This way, a vast number of complex samples can be tested and narrowed to the most promising ones to undergo further specific testing. 

## 2. Materials and Methods

### 2.1. Bacterial Strains and Culturing Conditions

A panel of six *E. coli* strains of bioluminescent bacteria was used to detect the potential antimicrobial properties of mushroom extracts (Table 1). The bacteria comprising this panel were genetically modified and contain different promoters fused with the lux operon, producing a quantifiable signal in the presence of several types of stressors.

The strains were selected for their wide detection capabilities, such that the whole panel can detect quorum sensing inducing compounds, heat shock, genotoxicity, fatty acid biosynthesis pathway interruption, and oxidative stress.

The bacteria were cultivated on Luria Bertini agar Difco (244520) supplemented with antibiotics to maintain pure cultures. Kanamycin sulfate (K1377-5G) was used for DPD1718 and K802NR strains, and ampicillin (A9518-5G) for strains TV1061, DPD2794, DPD2544, and DPD2511. The cultured plates containing 100 µg/mL of the ampicillin and 50 µg/mL of kanamycin were incubated at 37 °C for 24 h and then stored at 4 °C for further use. Cultures maintained their plasmid replication for up to 30 days, after which they were refreshed. Prior to antimicrobial compound screening, cultures were grown in 10 mL Luria-Bertani (LB) Broth Difco (244629) medium supplemented with the appropriate antibiotic for 12 h at 37 °C with shaking at 150 RPM (Innova44 Incubator Shaker Series, New Brunswick, NJ, USA). A secondary culture was prepared for each experiment by growing bacteria in an antibiotic-free LB medium for 2–3 h without shaking. Bacterial concentration was monitored using a spectrophotometer at 600 nm (Ultrospec 2100 Pro, Amersham, UK). Cultures reaching an optical density of 0.2 (approximately 10^6^ cfu/mL) were suitable for the assay since they are the most active at this stage. This specific stage within the exponential growth phase was crucial to avoid the need for dilutions.

### 2.2. Mushroom Extracts

Mushrooms were collected in Serbia in the period 2020–2023, except for the commercial preparations and *Inonotus obliquus*, which was collected in 2019. The extracts were prepared according to the procedures given in the literature (Table 2), using proprietary technology (PT), or as described further.

*Amanita muscaria* (tincture, 40% ethanol): the traditional procedure for tincture preparation was used. Fruit bodies were collected in July 2022 and cleaned from the soil. Only good-looking specimens (without blemishes or rotting sections) were used for tincture preparation. Only caps were used, cut into quarters, and soaked in 50% ethanol, a drug to solvent ratio of 1:3. For 40 days, the mixture was hand-shaken for about one minute. The liquid fraction was strained and filtered through a muslin cloth, and the alcohol content was measured and set to 40%.

Chaga (*Inonotus obliquus*) commercial dry extract: sublimated extract, CибПpибop, Irkutsk, Russia.

Chaga (*Inonotus obliquus*), tincture, 16% ethanol: dry and powdered mushroom was mixed with 50% ethanol in a 1:3 drug-to-solvent ratio and sonicated for one hour. The liquid was strained using a muslin cloth and then Whatman No. 4 paper. The filter cake was mixed with distilled water (three times the volume of ethanol) and slowly cooked for six hours (constantly boiling). After filtering through a gauze and being cooled to room temperature, the liquid part was mixed with the previously separated ethanol fraction and set to 16% *v*/*v* of ethanol. Before the experiment, ethanol was removed by rotary evaporator and dried at 40 °C.

*Cordyceps militaris*, *Ganoderma lucidum*, *Hericium erinaceus*, tincture, 26% ethanol: 25 g of dried and powdered fruit bodies were sonicated for 50 min together with 400 mL of ethanol (70%). The liquid part was then separated using Whatman No. 4 filter paper and kept separately. The leftover cake was transferred to an autoclave and cooked for 45 min with distilled water (500 mL) at 120 °C, 1.2 bar. After filtering through the muslin cloth, the liquid part was mixed with the previously prepared ethanol fraction and adjusted to 26% *v*/*v* strength of ethanol.

Ganoderic acids, crude extract in 70% ethanol: dried fruit bodies were pulverized into a fine powder; 20 g were mixed with 350 mL of 70% ethanol and sonicated in an ultrasound bath for 1 h. The mixture was filtered using Whatman No. 4 filter paper, and the liquid part presented crude ganoderic acid extract.

*G. lucidum* spores (cell-wall broken): proprietary procedure of collecting and processing spore powder, Ganoherb, Fujian Xianzhilou Biological Science and Technology Co., Ltd., Fuzhou, Fujian, China.

*H. erinaceus*, *Inonotus obliquus*, *Phellinus linteus*, *Fomitopsis betulinus*, *Pycnoporus cinnabarinus*, 70% ethanol: 5 g of dried and powdered mushrooms were mixed with 100 mL of 70% ethanol and sonicated for 45 min. Whatman No. 4 was used to separate the liquid part, which was further evaporated and dried at 40 °C.

*Ophiocordyceps sinensis*-mycelium, capsules, powder, standardized to 30% polysaccharides: HemoVital (ESENSA brand, Belgrade, Serbia).

Reishi (*G. lucidum*—PT) tincture, 26% ethanol: this sample was prepared in a way that resembles the traditional method of mushroom tincture preparation. Namely, a 650 mL jar was filled with dried *G. lucidum* chunks (3–4 cm in size) up to 2/3 of the jar’s volume and then filled with 42% ethanol so that chunks are covered with liquid. The jar was incubated for 2 months with vigorous daily shaking by hand for one minute. The liquid part was then strained through a gauze while chunks were cooked for five hours with three times the volume of the previously used ethanol. After leading it to a boil, the cooking temperature was decreased to allow steady boiling. Finally, the liquid was strained through a gauze, let to cool off, and mixed with the previously separated ethanol part and adjusted to 26% *v*/*v* of ethanol.

For each extract, the solvent was removed using a rotary evaporator (Heidolph Laborota 4000 Series, Schwabach, Germany) and dried in a laboratory drier at 40 °C. Finally, it was pulverized in mortar until fine powder was obtained. All mushroom extracts were solubilized in double-distilled water (DDW) or 0.25% dimethyl sulfoxide (DMSO) to ensure complete dissolution and accessibility to the bacterial plasmids. Table 2 lists all samples, including the species of foraged mushrooms, extraction methods, and solvents used for dissolution. Each sample was prepared as serial dilutions, ranging from 0.15 mg/mL to 10 mg/mL, in preparation for the bioassay.

### 2.3. Bioluminescence Kinetics

A Luminoskan Ascent luminometer (Thermo Fisher Scientific, Waltham, MA, USA) was used to measure bioluminescence kinetics. Bacteria were exposed to the mushroom extracts in white, opaque 96-well microtiter plates (Nunc, Roskilde, Denmark). Each well contained 90 µL of bacterial culture and 10 µL of the diluted mushroom sample. The arrangement of the plate wells started with blank samples containing only LB medium, a negative control as DDW or 0.25% DMSO, two concentrations of known inducers for the specific strain (Table 1), and mushroom extracts. Samples were tested in triplicate. Bioluminescence kinetics were measured over 20 h at 5-min intervals, resulting in 240 measurements per experiment. Each strain has a different total reaction time. Strain DPD1718 continues to produce bioluminescence after 24 h, while the other strains stop after 12 h.

### 2.4. Interpretation of Bioluminescence Kinetics

Bioluminescence kinetics in these bioreporters can be interpreted in two ways; induction of bioluminescence at low concentrations and inhibition of light production at higher concentrations. It is necessary to determine which dominates and draw conclusions on the antimicrobial effect and the specific pathway activation. This was done by testing several dilutions of examined mushroom extracts.

Every experiment has two controls, DDW and a known inducer. DDW serves as a negative control, and it shows the reaction of bacteria in a stress-free state. The inducers serve as positive controls, which are known to produce high bioluminescence by activating the promoters fused with the lux operon. This way, the response of the plasmid can be checked. DMSO 0.25% was also tested since it served as a solvent for some samples. It did not induce any bioluminescence in any of the strains, thus not influencing the results.

The induction factor (IF) was calculated based on bioluminescence kinetics and represents the ratio between the maximum bioluminescence of the sample and the maximum bioluminescence of the negative control (DDW or DMSO 0.25%):

$$\mathrm{IF}=\frac{MAX\;bioluminescence\;sample}{MAX\;bioluminescence\;control\;}$$

It shows the level of intensity a sample has compared to the control. When the induction factor is higher than 1.5, it signifies more than 50% increase in light response of the bacteria to the screened sample, compared with DDW. This value was considered the first threshold for toxicity evaluation. Further, if the induction factor is higher than 2, it represents twice the increase of bioluminescence, and this value was considered as the second threshold of toxicity. Besides those values, an inhibition of bioluminescence is also possible, and a value threshold was set to 0.5, meaning a 50% decrease in light emission. Samples were later classified based on calculated threshold values.

## 3. Results and Discussions

Diverse toxicity profiles among the tested mushroom extracts were observed. Extracts exhibited varying degrees of activity against quorum sensing molecules, heat shock (protein damage), genotoxicity, fatty acid biosynthesis inhibition, and oxidative stress. Based on the values of the induction factor, the results were placed into three groups: induction of bioluminescence, no effect, and inhibition. The induction of bioluminescence was further assigned a threshold: high (bioluminescence increased more than twice compared to control), low (up to twice the increase in bioluminescence compared to control), and distinct, where a specific concentration induced the highest bioluminescence. These values were assigned based on the induction of bioluminescence achieved with the positive controls. The concentration of positive controls for each of the strains (Table 1) induced an approximately 50% increase in light production. By comparison, mushroom samples that induced an induction factor higher than 2 were assigned “high” toxicity.

The following insights were discussed based on the experimental data, providing some understanding into the possible mechanisms of action the samples have on the bacteria.

**Figure 2:**
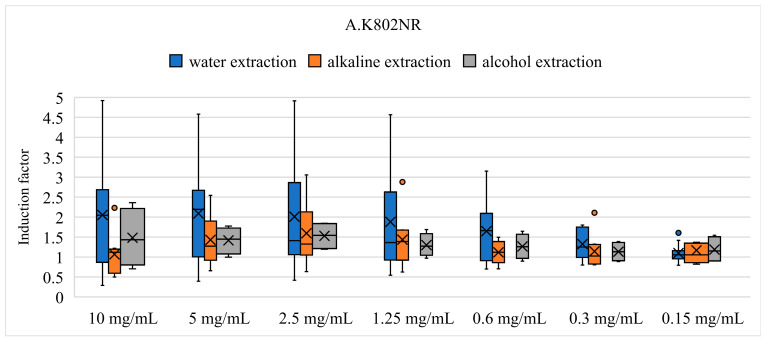

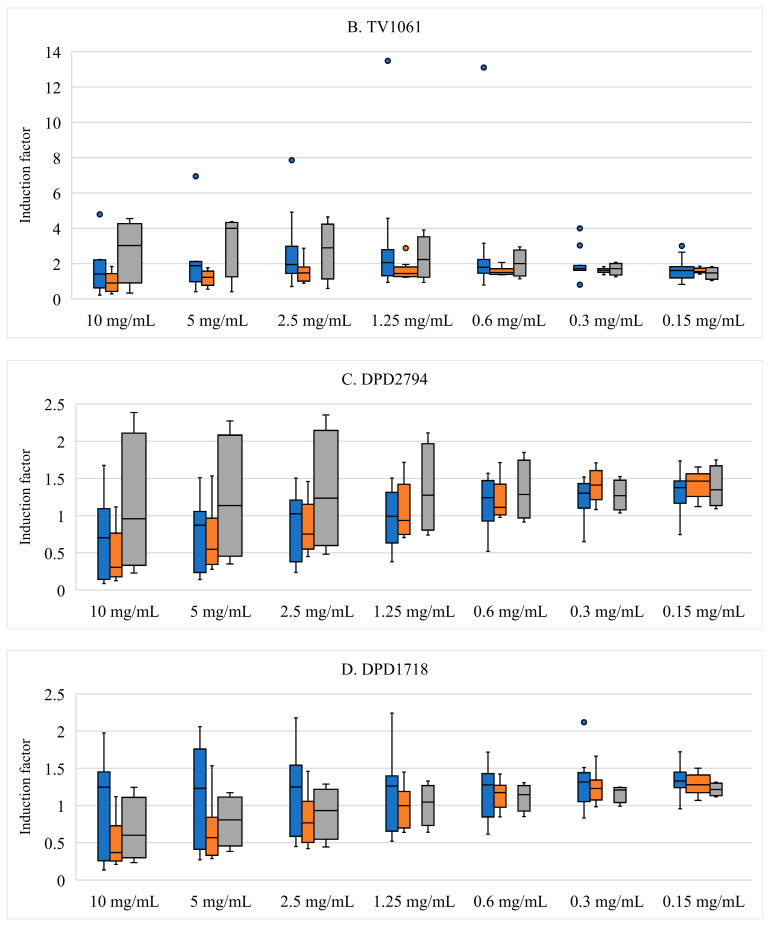

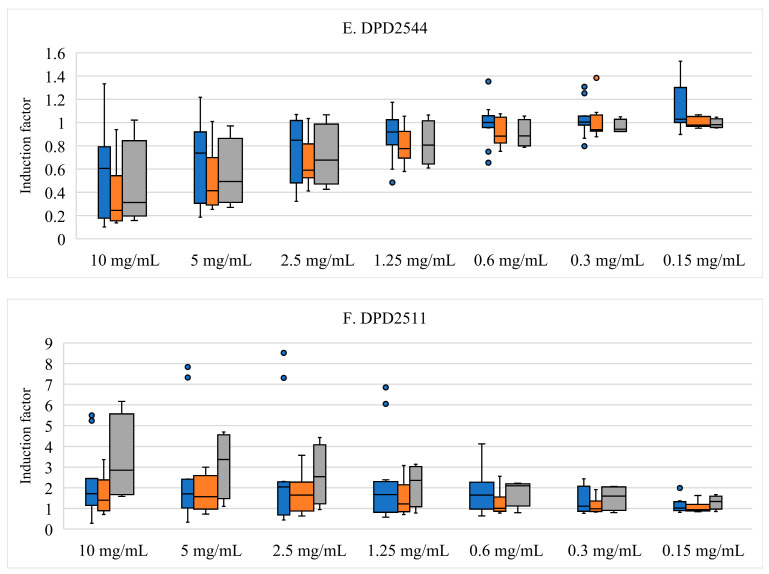
Box plots of the 11 samples and comparison between methods of extraction after exposure of strain K802NR (**A**), TV1061 (**B**), DPD2794 (**C**), DPD1718 (**D**), DPD2544 (**E**), and DPD2511 (**F**).

**Figure 3:**
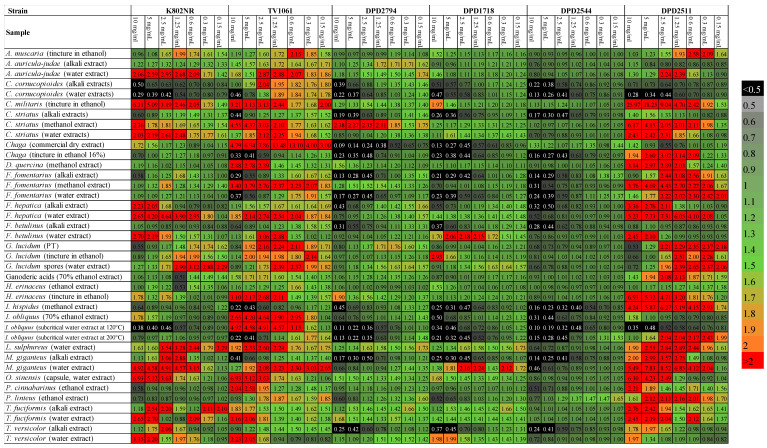
Heat map of induction factors after exposure of the bacteria to mushroom extracts. Black color signifies inhibition, green no effect, and red induction of bioluminescence.

(a) Selection of samples expressing a specific activity: by calculating the induction factor, it is possible to identify samples that are inducing bioluminescence (IF ≥ 1.5) and those that are inhibiting the bioluminescence IF ≤ 0.5 (Table 3 and Supplementary Material, Table S1: Individual profile toxicity).
(b) Concentration-bioluminescence relationship: the presence of linearity between the two variables was examined (Table 3).
(c) Correlation between method of extraction and IF: extracts were prepared by different extraction techniques, including different solvents, which enabled the examination of the influence of extraction and, thus, the chemical composition of the extract on tested bacteria (Figure 2).
(d) Active concentration: the correlation between the concentration of a sample and the IF was investigated (Figure 3).

**Table 3:** Summary of the results on toxicities.

Strain Sensitivity		Quorum Sensing	Heat Shock	Genotoxicity		Fatty Acid Biosynthesis Inhibition	Oxidative Stress
Strain Name		K802NR	TV1061	DPD2794	DPD1718	DPD2544	DPD2511
Promoters		*Iasl*	*grpE*	*recA*	*recA*	*fabA*	*katG*
No.	Name (Extraction)						
1	*A. muscaria* (tincture in ethanol)	+ *	++ *	No	+ *	No	++ *
2	*A. auricula-judae* (alkali extract)	No	+	+	No	No	No
3	*A. auricula-judae* (water extract)	++	++ *	+	No	No	++ *
4	*Chaga* (commercial dry extract)	+	+++	-	-	No	No
5	*Chaga* (tincture in ethanol 16%)	No	-	-	-	-	+++ *
6	*C. striatus* (alkali extracts)	No	+	-	-	-	+ *
7	*C. striatus* (methanol extract)	++	+++	++	No	No	+++
8	*C. striatus* (water extracts)	++	+	No	+ *	No	++
9	*C. militaris* (tincture in ethanol)	+++	+++	+ *	+ *	No	+++
10	*C. cornucopioides* (alkali extracts)	No	+	-	No	-	No
11	*C. cornucopioides* (water extracts)	-	+	-	-	-	-
12	*D. quercina* (methanol extract)	No	++	+	No	No	++
13	*F. hepatica* (alkali extract)	++	+	-/+	No	-	+++
14	*F. hepatica* (water extract)	+++	++	+	No	No	+++
15	*F. fomentarius* (alkali extract)	+ *	+	-/+	-	-	++ *
16	*F. fomentarius* (methanol extract)	+ *	+++	+ *	No	-	+++ *
17	*F. fomentarius* (water extract)	No	+	-	-	-	++
18	Ganoderic acids (70% ethanol extract)	No	+	No	No	No	++ *
19	*F. betulinus* (alkali extract)	No	+	-	-	-	No
20	*Fiptoporus betulinus* (water extract)	+	++ *	No	++ *	No	++
21	*G. lucidum* (PT)	+	++	+	No	No	++ *
22	*G. lucidum* (tincture in ethanol)	+	++ *	No	++ *	No	++ *
23	*G. lucidum* spores (water extract)	+++*	++	+	+	No	++ *
24	*H. erinaceus* (ethanol extract)	+ *	+++ *	No	No	No	+++
25	*H. erinaceus* (tincture in ethanol)	+ *	+ *	+	+ *	No	No
26	*I. hispidus* (methanol extract)	No	-	-	-	-	+++ *
27	*I. obliquus* (70% ethanol extract)	+	+++ *	No	No	-	+
28	*I. obliquus* (subcritical water extract at 120 °C)	-	+++	-	-	-	-
29	*I. obliquus* (subcritical water extract at 200 °C)	No	+	-	-	-/+	++
30	*L. sulphureus* (water extract)	+++ *	++	+	+	No	++ *
31	*M. giganteus* (alkali extract)	+++ *	-	-	-	-	++ *
32	*M. giganteus* (water extract)	+++	+++	No	++ *	-	+++ *
33	*O. sinensis* (capsule, water extract)	+++	+++	+	+	No	+++
34	*P. linteus* (ethanol extract)	No	+	+	No	+	++*
35	*P. cinnabarinus* (ethanol extract)	No	++ *	No	No	No	++
36	*T. versicolor* (alkali extract)	++ *	No	-	-	-	+
37	*T. versicolor* (water extract)	+++	++	+ *	+ *	No	+
38	*T. fuciformis* (alkali extract)	++ *	+	+ *	+ *	No	++
39	*T. fuciformis* (water extract)	++ *	++	+ *	No	No	++

Legend: “+” IF > 1.5 (>50% light increase), “++” IF > 2(>2x light increase), “+++” IF > 3 (>3× light increase), “-” IF < 0.5, (<50% light decrease), “No” no effect, “*” distinct toxicity (not dose-dependent response), “-/+” both induction and inhibition depending on the concentration.

Furthermore, the comparison between the methods of extraction and the induction factor is shown for 11 different mushroom species. Different results were obtained from the three different methods of extraction. The following box plots show the difference between the three methods of extraction. The main components are the box, whiskers (lines outside the box), the median inside the box, and outliers. The box is made from 50% of the data, divided by the median line, where its top goes up to 75% and the bottom down to 25%. The whiskers extend from the box to represent the data range within 1.5 times from the box. Last, the outliers show the values that are distributed far away.

### 3.1. Quorum Sensing (Strain K802NR, Promoter Iasl)

*E. coli* strain K802NR is a spontaneous antibiotic-resistant mutant (nalidixic acid, rifampin) derived by sequential selection [39,48]. It contains the plasmid pSB1075, which is a fusion between the promoter *lasRI* and the *lux operon CDABE* that is activated by a transcriptional response to the presence of long-chain acyl-homoserine lactone (AHL) [49]. AHL are a class of intercellular compounds involved in the cell density-dependent gene regulation between Gram-negative bacteria and represent a chemical communication method called quorum sensing [50]. These compounds diffuse out through the membranes and accumulate in the surrounding environment of the bacteria. Once they reach a critical concentration, collective gene expression occurs [51], such as coordinating phenotypic activities, including biofilm formation, virulence, conjugation, antibiotic secretion, rhamnolipid synthesis, and cell motility [52,53,54,55,56,57]. The sensitivity of this strain to quorum sensing results in light production when communication between cells is affected by exogenous compounds. These compounds, usually small molecules, may have contrasting effects on the bacterial metabolism depending on their concentration, by either altering the transcription patterns or inhibiting the growth by suppressing specific target functions [58]. The detection of pharmaceutically useful natural product inhibitors can be achieved by measuring transcription activation at low concentrations in this strain.

Based on the IF of different mushroom extracts, 32 out of 39 samples had a significant effect on this strain (Figure 3). This is of particular importance since it confirms that most mushrooms (the kingdom of fungi) tested here affect quorum sensing and thus the way bacteria coordinate biological functions like virulence factor production [59]. Orio et al. [60] reported the existence of dynamic interaction between bacteria and fungi based on secreted molecules, like AHL, which is also observable from a wide range of luminescence responses in our study.

The criteria for this analysis strictly separated samples into groups based on their induction factor as previously described (2.4). Induction of bioluminescence was achieved by 27 samples, where *O. sinensis* (capsule, water extract) had seven times the bioluminescence compared with control, followed by *C. militaris* (tincture, 26% *v*/*v* ethanol) with 6.3 times the bioluminescence. Four samples inhibited the production of bioluminescence, all at 10 mg/mL (*C. cornucopioides* (water and alkali extract), *I. obliquus* (subcritical water extract at 120 °C), and *P. cinnabarinus* (ethanol extract). The lowest tested concentration (0.15 mg/mL) had minimal to no effect on K802NR no matter which sample was tested. The correlation between the induction factor and concentration is presented in Table 3, where samples that did not induce a dose-dependent response are marked with an *. The majority of extracts showed a linear trend; the higher the concentration, the higher the bioluminescence. Samples extracted in water induced higher bioluminescence than other extraction methods, with two exceptions being *F. fomentarius* and *I. obliquus* (Figure 2A).

As demonstrated by several authors, the decrease in bioluminescence, expressed as IF, indicates that the tested substance exhibits potential antimicrobial activity [61,62]. In the case of water and alkali extracts of *C. cornucopioides* tested against Gram-negative bacteria, *Salmonella enteritidis*, the same concentration (10 mg/mL) has been reported as the minimum inhibitory concentration (MIC) [45]. Similarly, subcritical water extracts of *I. obliquus* tested against several Gram-negative species showed inhibition of bacterial growth at 20 mg/mL (unpublished results), again confirming the results obtained with bioreporter bacteria. These extracts (especially water and alkali) mainly consist of polysaccharides (α- and β-glucans) with lesser amounts of small molecules like phenolic compounds (especially in alkali extracts due to more aggressive alkaline destruction of fungal cell walls) and protein fragments as a consequence of the extraction method [63]. However, mushroom extracts, especially crude polysaccharide extracts, exhibit antibiotic-like and anti-quorum sensing activity against Gram-positive and Gram-negative pathogenic bacteria [64,65].

On the other hand, most of the samples induced an increase in bioluminescence. Although the intuitive conclusion can lead to explaining this behavior as samples acting as stimulators of quorum sensing, thus even bacterial growth and biofilm formation, the literature indicates no straightforward or clear answer. For example, Thorn et al. [66] discussed that in a killing environment, cells produce more light. This statement is also supported by the previous study showing that genetically constructed strains produce more light under stress conditions due to the activation of survival mechanisms [67]. We speculate that mushrooms stimulate quorum sensing in bacterial communities, which triggers different responses, such as modulation of metabolic processes when applied in subinhibitory concentrations, as previously stated by Goh et al. [58]. When the MIC of the same samples, reported previously by Vunduk et al. [45], is compared with concentrations that induced the highest IF, it is evident that concentrations reported in this study are below MIC values. A method of extraction-wise water and alkali extraction gave samples that mostly increased IF, especially hot water extracts presenting a combination of polysaccharides of higher molecular weights and low amounts of phenolics and proteins.

At the same time, hot alkali extraction produces extracts with more degraded polysaccharides. It can be speculated that high molecular weight polysaccharides activate bacterial communication at a higher grade, while poly/oligosaccharides (alkali extracts) exhibited antimicrobial activity. This has also been demonstrated by Klaus et al. [63,68,69]. When it comes to the source of extracts, *O. sinensis* (previously known as *Cordyceps sinensis*) and *C. militaris* induced the highest increase of bioluminescence, significantly higher than any other sample. This leads to the conclusion that the “toxic” effect on bacterial communities strongly depends on genus and not exclusively individual species. Previous research on mushroom extracts on quorum sensing activity showed an overall inhibition of the production of bioluminescence in several strains of bacteria, which was addressed to the total phenolic compounds found in mushrooms [70,71], polysaccharides [63], and possibly to other secondary metabolites [72]. This suggests that some mushroom species may have a potential role in disrupting quorum sensing and could possibly be used instead of or in combination with antibiotic compounds [73,74,75,76]. The assessment of quorum sensing activity by the strain K802NR used in this research proved to be an effective way to screen mushroom extracts for possible utilization as antimicrobial compounds.

### 3.2. Heat Shock (Strain TV1061, Promoter grpE)

*E. coli* strain TV1061 contains a plasmid-borne fusion of the *grpE* promoter to the *lux operon CDABE* [40]. The promoter used in this strain is activated by the induction of heat shock, which is induced by a variety of environmental stresses, including protein-damaging compounds and organic molecules, among others [77,78]. Its utility has been as an excellent general toxicity sensor [78]. Besides the multi-copy plasmid, the strain also contains a mutation of the outer membrane, tolC, which enhances the detection of hydrophobic molecules by allowing easier diffusion into the cytoplasm [40]. This strain is particularly useful for screening natural products due to its ability to sense organic molecules inducing protein damage [79]. To identify the meaning of toxicity, both low and high concentrations of an extract must be analyzed and then determined as to which is more significant.

All samples affected this strain (Figure 3). More precisely, three extracts (*I. hispidus* (methanol extract), *Chaga* (tincture in ethanol, 16%), and *M. giganteus* (alkali extract)) inhibited the production of bioluminescence significantly at low concentrations (0.15 mg/mL and 0.6 mg/mL, respectively). This means that the effect of the mentioned samples was direct, an intense heat shock resulting in bacteria’s death. Moreover, these samples did not induce anti-quorum sensing in K802NR, pointing to the fact that the mechanisms by which fungi affect bacteria are diverse and species-specific. Also, the samples were prepared using different extraction methods, thus leading to the speculation that compounds present in very small quantities might be responsible for the observed effect. Four samples induced high bioluminescence at low concentrations while inhibiting the production of bioluminescence at 10 mg/mL (*C. striatus* (alkali extracts), *I. obliquus* (supercritical water extract at 200 °C), *C. cornucopioides* (water extracts), and *F. fomentarius* (water extract). The remaining extracts, 32 in total, induced significant production of bioluminescence. This confirms the strong relationship between fungi and bacteria; all samples we tested produced reactions. One sample, *Chaga* (commercial dry extract), induced 13 times the bioluminescence compared to the control (at only 1.25 mg/mL), while 10 mg/mL had only 4.7 times the bioluminescence and 0.15 mg/mL had just three times the bioluminescence. Interestingly, the concentration of 1.25 mg/mL was the maximum one inducing bioluminescence. Thus, the toxic effect cannot be generalized and must be thoroughly examined for each species since the same sample can exhibit diverse activity with the threshold value at which the disturbance effect becomes lethal.

In general, mushroom extracts showed a positive correlation between concentration and IF (Table 3). There were nine exceptions, the most significant of which was *H. erinaceus* (tincture in ethanol), which exhibited a 50% increase in bioluminescence at 0.6 mg/mL. Samples extracted in alcohol induced higher bioluminescence than other extraction methods, followed by water extraction (Figure 2B). Inhibition of bioluminescence was more significant in samples undergoing alkaline extraction. Extraction method-wise, it would seem that an aggressive alkali protocol resulted in extracts with more lethal chemical compositions. This has been previously reported by Klaus et al. [63] when the antimicrobial activity of differently prepared extracts of *Grifola frondosa* was tested. The authors demonstrated that alkali extraction causes the break of glycosidic linkages, resulting in a mixture of poly/oligosaccharides [68,69]. On the other hand, water extracts present a more diverse mixture of polysaccharides, proteins, and aromatic compounds. Samples prepared using ethanol increased bioluminescence, possibly due to the presence of phenolic compounds, which are also responsible for the antioxidant activity of mushroom extracts. They might act as scavengers or pro-oxidants, depending on the concentration and conditions, thus provoking oxidative stress in bacteria [80]. This can cause damage resulting in cell death or a state of alert in bacteria, here represented by the bioluminescence increase [81].

Several types of terpenes isolated from mushrooms proved to have a cytotoxic effect [82,83,84,85]. While the mechanisms are varied, we showed it is possible to determine cytotoxicity by measuring the heat shock response of strain TV1061. Prescreening of natural products in drug discovery was reported in other research using the same strain [86].

### 3.3. Genotoxicity

Both strains (DPD2794 and DPD1718) contain the same promoter, *recA* fused to the *lux* operon, with the main difference being in the location of the gene complex. DPD2794 has a multi-copy plasmid with the mentioned gene complex [41], while DPD1718 has the gene complex inserted into the bacterial chromosome [17]. While both strains respond to the same type of stress, their response differs in terms of time of induction, intensity, and sensitivity. They allow for rapid measurement of sub-lethal concentrations of DNA-damaging compounds.

#### 3.3.1. Strain DPD2794 (Promoter *recA*)

The induction of bioluminescence starts in the first hour of exposure of the bacteria to the sample and can last up to four or five hours. The plasmid contained in this strain allows for real-time reporting of the transcriptional activation of SOS and adaptive response-regulated operons [87,88].

Of all the extracts, 9 samples did not affect the bacteria, 14 samples inhibited the bioluminescence production, and 16 samples induced the production of bioluminescence (Figure 3). Two samples inhibited the production of bioluminescence at 10 mg/mL and, at the same time, induced the production of bioluminescence at 0.15 mg/mL (*F. fomentarius* (alkali extract) and *F. hepatica* (alkali extract). As evident, both samples were alkali extracts, in which, as already mentioned, poly/oligosaccharides are the dominant compounds, pointing to the fact that lower molecular weight sugars of mushroom origin can induce genotoxicity in bacteria when applied at higher concentrations.

In general, samples had a linear trend between concentration and induction factor, where lower concentrations induced a higher signal and higher concentrations inhibited the production of bioluminescence. This could imply that samples inhibiting the production of bioluminescence could have antimicrobial effects by exhibiting direct genotoxicity. There were five exceptions where only specific concentrations induced the production of bioluminescence up to two times. Interestingly, the extraction method did not prove to change the induction factor in this strain. Different concentrations resulted in the same induction factor (Figure 2C). In parallel, the samples that inhibited bioluminescence were mainly Polypore species, generally good sources of phenolic acids [46].

#### 3.3.2. Strain DPD1718 (Promoter *recA*)

*E. coli* strain DPD1718, the second strain in this panel that is able to detect genotoxicity, contains the fusion of promoter *recA* with the *lux* operon, inserted directly into the bacterial chromosome at the *lacZ* locus of *E. coli.* [42]. The sensitivity of this strain is higher than DPD2794; however, it can produce a signal for up to 20 h, with a homogeneous increase or decrease in intensity rather than a spike.

The results of the IF for the examined mushroom extracts yielded different results than for DPD2794, with more samples inhibiting the production of bioluminescence, thus exerting direct genotoxicity. In total, nine samples did not induce any effect on the bacteria; 15 samples inhibited bioluminescence, while 14 samples induced the production of bioluminescence (Figure 3). Only one sample inhibited the production of bioluminescence at 10 mg/mL while inducing it at 0.15 mg/mL (*F. hepatica*, alkali extract).

There was a linear correlation between the concentration of the samples and the induction factor, with the highest induction of 2.9 times that of the control. There are nine exceptions where only a specific concentration induced significant bioluminescence. Slight differences were observed between the methods of extraction and induction or inhibition of bioluminescence, and only three samples had a significant difference (Figure 2D).

The use of bioluminescent bacteria as a bioassay is a reliable method for the detection of sub-inhibitory concentrations of genotoxicity. Most of the mushroom samples had a low induction or inhibition of the strains used in this research; however, few samples were found to induce high bioluminescence as a response to the DNA repair system used by the bacteria.

### 3.4. Fatty Acid Biosynthesis Inhibition (Strain DPD2544, Promoter fabA)

*E. coli* strain DPD2544 contains the plasmid *pfabALux*, which has a fusion of the *fabA* promoter to the *lux operon CDABE* [43]. The promoter used in this strain is activated in response to a broad spectrum of toxicants which interrupt the general well-being of the cells. The mechanism by that these compounds affect the intracellular concentration of long-chain acyl-CoA molecules may be varied, as was reported by the induction of bioluminescence by alcohols, phenols, and derivatives, halomethanes, aromatics, and detergents [43].

Half of the total number of samples affected the bacteria, and half did not induce any significant effect. Most of the samples inhibited the production of bioluminescence, 17 samples out of 19, all at 10 mg/mL (Figure 3). This means that 17 samples reduced the bacterial population, probably by interfering with fatty acid biosynthesis. Two samples induced the production of bioluminescence, both at 0.15 mg/mL (*P. linteus*, ethanol extract and *F. betulinus*, water extract), and one sample, *F. hepatica* (water extract), inhibited the production of bioluminescence at 10 mg/mL and, at the same time, induced the production of bioluminescence at 0.15 mg/mL. In general, mushroom extracts showed a linear trend, with higher concentrations inhibiting the production of bioluminescence. There is only a slight difference in the inhibition of bioluminescence made by samples extracted in an alkaline solution (Figure 2E). These results show that one of the mechanisms of mushrooms’ antimicrobial activity may be the interruption of fatty acid synthesis, especially when higher concentrations are applied. This is in line with benzoic acid derivatives proposed as FASII enzyme (catalyzing fatty acid synthesis in most bacteria) inhibitors [89]. Benzoic acid derivates are often constituents of Basidiomycetes. For example, Szychowski et al. [90] stated that *I. obliquus* contains benzoic acid derivates, among other bioactive substances. In our study, differently prepared extracts of *I. obliquus* inhibited the production of bioluminescence, probably due to the presence of FASII enzyme inhibitors. On the other hand, mushrooms also contain sterols, which offer a protective activity against metabolism disturbances, explaining why some samples did not show any effect on this strain [91,92,93,94].

### 3.5. Oxidative Stress (Strain DPD2511, Promoter katG)

*E. coli* strain DPD2511 contains the pKatGLux2 plasmid, which has a fusion of promoter *katG* to the *lux operon CDABE* [44]. The plasmid used in this strain exhibits low basal levels of luminescence, which increase hundred to thousands of times in the presence of oxidative agents (such as peroxides, hydrogen peroxide, redox-cycling agents, and hydrogen peroxide-producing enzymes—xanthine and xanthine oxidase) and can be used as a tool for assaying oxidant and antioxidant properties of chemicals, quantifying the effects of these oxidative agents, and studying cellular responses to oxidative stress [44].

Of the total of 39 samples of mushroom extracts, four samples did not induce any significant effect on the bioreporters, two samples inhibited bioluminescence, and 32 samples induced bioluminescence. One sample (*G. lucidum*—tincture) inhibited the bioluminescence at 10 mg/mL and at the same time induced the production of bioluminescence at 0.3 mg/mL (Figure 3).

In general, there was a correlation between the concentration of a sample and IF; the higher the concentration, the higher the bioluminescence. However, there are 15 exceptions where only specific concentrations induced significantly the production of bioluminescence. The difference between the extraction methods and the induction factor can be observed in Figure 2F. A different chemical profile due to the different extraction method resulted in different induction factor values.

As for oxidative stress, strain DPD2511 is an excellent tool for determining oxidative agents. Many samples in this study showed some kind of oxidative stress, as shown previously from the calculated induction factors. Opposite to the omnipresent opinion that mushrooms are excellent sources of antioxidants for humans, most samples presented here acted as oxidants in the presence of a DPD2511 mutant. Organic peroxides, like ergosterol peroxide [95] present in *G. lucidum* as well as in other medicinal mushrooms, might induce damage to the membrane or cytoplasm of the bacterial cells. In our study, the highest value of bioluminescence induction has been observed for *C. militaris* prepared with ethanol. Recently, members of the *Cordyceps* genus have been shown to contain ergostane-type sterols with biological properties [96].

Crude extracts present a complex mixture of biologically active molecules with often contrasting effects. As observed in our study, for each extract, the observed effect upon whole-cell bioreporter bacteria depends on the fine balance between oxidant and antioxidant compounds. Thus, purification and more research targeting specific needs, both in vitro and in vivo, should be performed to draw final conclusions.

## 4. Conclusions

The proposed bacterial panel bioassay provides valuable insight into the possible antimicrobial mechanisms of mushroom extracts, which currently cannot be obtained by any other test. It allows for high-throughput screening of a large number of samples while decreasing the time needed for initial results in their biological mechanisms. Most samples exhibited a light induction response in at least one mutant strain. The bioluminescent reporter strain K802NR, sensitive to quorum sensing disruption, revealed that a significant majority of the tested mushroom extracts (32 out of 39) affected bacterial cell-to-cell communication. This finding highlights the potential of mushrooms as a rich source of quorum sensing modulators, with particular emphasis on the extracts of *Ophiocordyceps sinensis* and *Cordyceps militaris*, which exhibited the most potent quorum sensing-inducing activities. The heat shock-detecting strain TV1061 indicated that many mushroom extracts can induce cellular stress responses in bacteria, with both stimulatory and inhibitory effects observed. This suggests that mushroom-derived compounds can elicit diverse mechanisms of action, including the potential for antimicrobial activity through the disruption of protein homeostasis. The genotoxicity assays using strains DPD2794 and DPD1718 revealed that several mushroom extracts, particularly those obtained through alkaline extraction, can induce DNA damage in bacteria. This highlights the importance of considering extraction methods when evaluating the bioactivity of mushroom-derived compounds. The fatty acid biosynthesis inhibition assay with strain DPD2544 demonstrated that 17 samples, especially those with higher concentrations, can disrupt bacterial lipid metabolism, likely through the inhibition of fatty acid synthesis enzymes. Lastly, the oxidative stress sensor strain DPD2511 indicated that most mushroom extracts exhibited oxidant activities on the cells. This highlights the complex nature of the bioactive properties of crude mushroom extracts. Extraction technique-wise, hot water extraction resulted in samples that exhibited the strongest bioluminescence, while the relationship between the concentration and the signal was not always linear. The explanation of mushrooms’ mechanisms of antimicrobial activity can be enhanced and tailored by modifying the test panel. Due to this modular option, the whole-cell bioreporter bacteria panel is flexible and fits the needs of specific industrial or market requirements as well as different materials (for example, plant extracts). Moreover, it can be combined with traditional techniques for more in-depth and comprehensive analysis.

## Figures and Tables

**Table 1:** Panels of bioluminescent bacteria used in this research.

*E. coli* Strain	Plasmid/Host	Promoter	Strain Sensitivity	Inducer of Bioluminescence	Conc. of Inducer	Ref.
K802NR	pSB1075/K-12	*Iasl*	Quorum sensing	Nalidixic acid	0.1 mg/mL	[39]
TV1061	pGrpELux/RFM443	*grpE*	Heat shock	Ethanol	15.8 mg/mL	[40]
DPD2794	pRecALux/RFM443	*recA*	DNA damage	Mitomycin C	0.0008 mg/mL	[41]
DPD1718	*/DPD1692	*recA*	DNA damage	Mitomycin C	0.0008 mg/mL	[42]
DPD2544	pFabALux/W3110	*fabA*	Fatty acid biosynthesis inhibition	Phenol	0.047 mg/mL	[43]
DPD2511	pKatGLux2/RFM443	*katG*	Oxidative stress	Hydrogen peroxide	0.0035 mg/mL	[44]

* Promoter and *lux* operon inserted into the bacterial chromosome. Sources of bacterial strains can be found in the acknowledgements section.

**Table 2:** List of mushrooms and the solvents used for extraction.

Nr.	Name	Initial Form	Extraction	Extraction Ref.	Solvent for Extract
1	*Amanita muscaria*	tincture, 40% *v*/*v* ethanol	ethanol	PT	0.25% DMSO
2	*Auricularia auricula-judae*	dry extract	alkaline	[45]	water
3	*Auricularia auricula-judae*	dry extract	water	[45]	water
4	*Chaga* (*Inonotus obliquus*)	commercial dry extract	water	PT	water
5	*Chaga* (*Inonotus* *obliquus*)	tincture, 16% *v*/*v* ethanol	ethanol	PT	0.25% DMSO
6	*Ciatus striatus*	dry extract	alkaline	[45]	water
7	*Ciatus striatus*	dry extract	methanol	[46]	0.25% DMSO
8	*Ciatus striatus*	dry extract	water	[45]	water
9	*Cordyceps militaris*	tincture, 26% *v*/*v* ethanol	combined water and ethanol	PT	0.25% DMSO
10	*Craterellus cornucopioides*	dry extract	alkaline	[45]	water
11	*Craterellus cornucopioides*	dry extract	water	[45]	water
12	*Daedalea quercina*	dry extract	methanol	[46]	0.25% DMSO
13	*Fistulina hepatica*	dry extract	alkaline	[45]	water
14	*Fistulina hepatica*	dry extract	water	[45]	water
15	*Fomes fomentarius*	dry extract	alkaline	[45]	water
16	*Fomes fomentarius*	dry extract	methanol	[46]	0.25% DMSO
17	*Fomes fomentarius*	dry extract	water	[45]	water
18	*Fomitopsis betulinus*	dry extract	alkaline	[45]	water
19	*Fomitopsis betulinus*	dry extract	ethanol		water
20	Ganoderic acids, extract	70% *v*/*v* ethanol extract, crude	ethanol	PT	0.25% DMSO
21	*Ganoderma lucidum*	tincture, 26% *v*/*v* ethanol	combined water and ethanol	[45]	0.25% DMSO
22	*Ganoderma lucidum* (PT)	tincture, 26% *v*/*v* ethanol	combined water and ethanol extract	PT	0.25% DMSO
23	*Ganoderma lucidum* spores (cell wall broken)	dry extract, commercial	proprietary technology	PT	water
24	*Hericium erinaceus*	dry extract	ethanol, 70%	PT	0.25% DMSO
25	*Hericium erinaceus*	tincture, 26% *v*/*v* ethanol	combined water and ethanol extract	[45]	0.25% DMSO
26	*Inonotus hispidus*	dry extract	methanol	[46]	0.25% DMSO
27	*Inonotus obliquus*	dry extract	70% ethanol		water
28	*Inonotus obliquus*	dry extract	subcritical water extract at 120 °C	[47]	water
29	*Inonotus obliquus*	dry extract	subcritical water extract at 200 °C	[47]	water
30	*Laetiporus sulphureus*	dry extract	water	[45]	water
31	*Meripilus giganteus*	dry extract	alkaline	[45]	water
32	*Meripilus giganteus*	dry extract	water	[45]	water
33	*Ophiocordyceps sinensis*-mycelium	capsules—dry extract powder (standardized to 30% polysaccharides)	water	PT	water
34	*Phellinus linteus*	dry extract	ethanol		water
35	*Pycnoporus cinnabarinus*	dry extract	ethanol		0.25% DMSO
36	*Trametes versicolor*	dry extract	alkaline	[45]	water
37	*Trametes versicolor*	dry extract	water	[45]	water
38	*Tremella fuciformis*, cultivated	dry extract	alkaline	[45]	water
39	*Tremella fuciformis*, cultivated	dry extract	water	[45]	water

## Data Availability

All data related to this research may be found in our records in footprints (https://footprints-b291f.web.app/).

## Supplementary Materials

The following supporting information can be downloaded at: https://www.mdpi.com/article/10.3390/bios14110558/s1, Table S1: Individual profile toxicity.
